# Life-threatening Episodes of Malignant Hyperthermia Following Halothane Anesthesia in Three Children: A Case Series and Review of Literature

**DOI:** 10.5005/jp-journals-10071-23112

**Published:** 2019-01

**Authors:** Somrita Laha, Prabhas P Giri, Agnisekhar Saha, Partha P Gupta, Anisha De

**Affiliations:** 1-3 Department of Pediatric Intensive Care, Institute of Child Health, Kolkata, West Bengal, India; 4 Department of Pediatric Surgery, Institute of Child Health, Kolkata, West Bengal, India; 5 Department of Anaesthesia, Institute of Child Health, Kolkata, West Bengal, India

**Keywords:** Anesthesia, Dantrolene, Halothane, Malignant hyperthermia, Ryanodine receptor

## Abstract

Malignant hyperthermia (MH) is an inherited, pharmacogenetic disorder of the skeletal muscle, characterized by dangerous hypermetabolic state after anesthesia with succinylcholine and/ or volatile halogenated anesthetic agents, clinically manifested as hyperpyrexia and related complications like tachycardia, tachypnea, increased carbon dioxide production, increased oxygen consumption, acidosis, rigid muscles, rhabdomyolysis and disseminated intravascular coagulation (DIC). Here we present a series of three cases of MH, admitted in our hospital in a span of 8 months for three different operative procedures to be done under general anesthesia (cleft lip repair, Duhamel's operation for Hirschsprung's disease and surgical repair of development dysplasia of hip), who developed probable hyperthermia owing to Halothane being used as an anesthetic agent.

**How to cite this article:**

Laha S, Giri PP, Saha A, Gupta PP, De A. Life-threatening Episodes of Malignant Hyperthermia Following Halothane Anesthesia in Three Children: A Case Series and Review of Literature. Indian Journal of Critical Care Medicine, January 2019;23(1):47-50.

## INTRODUCTION

Malignant hyperthermia (MH) or malignant hyperpyrexia is a severe reaction under general anesthesia in susceptible individuals, first described in 1960 by Denborough.^1^ It is an autosomal dominant neuromuscular disease involving defects in calcium release, triggered mostly by inhalational anesthetic agents, and can be fatal if not treated promptly.^2^ In our experience, we faced three cases of probable malignant hyperthermia where the surgery went uneventfully, but the signs and symptoms suggestive of malignant hyperthermia appeared in the recovery room in all the patients. Immediate resuscitative measures were taken and mechanical ventilation done in all, two of them could be successfully revived and saved while the third died within a couple of hours. This case series discusses in detail the not so rare existence of malignant hyperthermia after halothane induction and the gravity with which it should be dealt with, especially due to nonavailability of dantrolene, the only specific therapy for MH in most of the places.

## CASE REPORTS

### Case 1

**Fig. 1:**
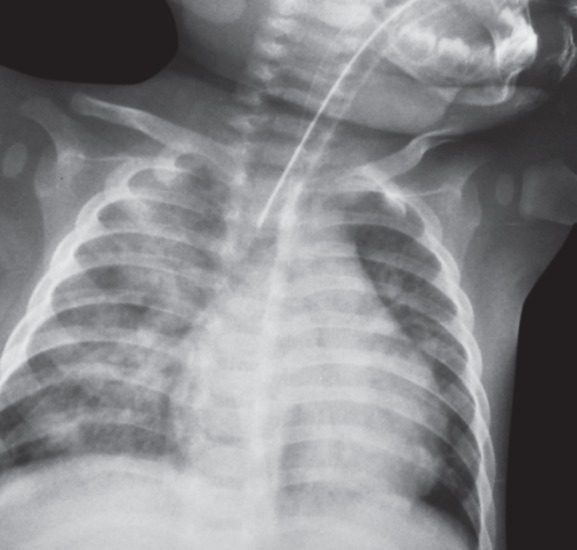
Chest X-ray of case 1 showing pulmonary hemorrhage

**Fig. 2:**
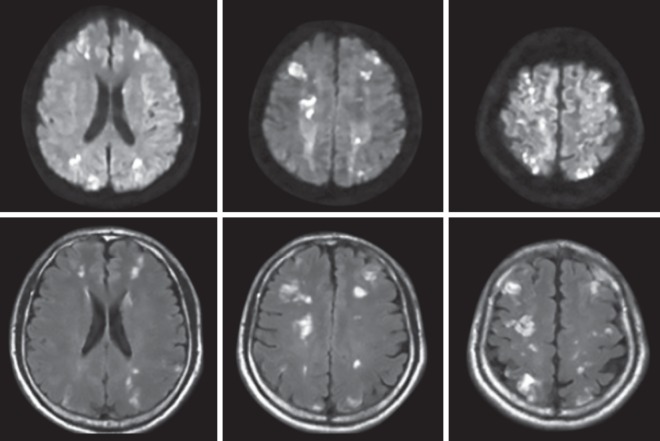
MRI brain of case 1 showing multiple infarcts

A 10-month-old male child was placed for repair of cleft lip and palate. Following uneventful surgery under GA where Halothane was used as an inhalational agent, he developed a high fever (107°F) and right-sided focal convulsion in the recovery room. Shifted to PICU with continuing convulsions, he also developed generalized hypertonia and hematuria along with decreasing urine output and increasing urea and creatinine. Serum creatine phosphokinase (CPK) was 15970 U/L. Treatment was commenced with hyperhydration and cold sponging. Next day, though renal function improved, hyperthermia continued with convulsions, rising CPK (>18000) and disseminated intravascular coagulation (DIC). In the face of poor GCS and deteriorating respiratory pattern, he was intubated and put on mechanical ventilation. Multiple units of FFP, platelet, and PRBC were transfused. Following 7 days of mechanical ventilation, he was extubated, only to be reintubated 2 days later, owing to secondary sepsis and profuse pulmonary hemorrhage and was again ventilated for 14 days (Fig. 1). Following extubation, he improved gradually but had severe developmental regression. MRI brain revealed multiple infarcts in the brain (Fig. 2). On follow up he gained his milestones up to a certain extent but was still having a global delay.

### Case 2

A 1-year-old male child was admitted for definitive repair of Hirschsprung's disease with a colostomy already in place since the neonatal period. The child underwent routine investigations and was operated under GA with Halothane, but the operation was unsuccessful. In the recovery room, just after 25 minutes of completion of the surgery, he developed high-grade temperature (105.8° F) followed by one episode of GTCS and was immediately shifted to PICU. He developed refractory status epilepticus with the irregular respiratory pattern, generalized hypertonia, after that to protect airway he was intubated and ventilated. Keeping the possibility of malignant hyperthermia in mind, relevant investigations were sent. He was loaded with multiple antiepileptic drugs, all possible neuroprotective strategies were taken, in spite of that repeated episodes of convulsion continued, and he succumbed to death after the third cardiac arrest after 14 hours of PICU admission. Blood reports revealed a CPK 16400 U/L, serum potassium 7 mEq/L, serum calcium mg/dL, with metabolic acidosis in the blood gas (pH 7.19, PCO_2_ 55 mm Hg, HCO_3_ 10 mEq/L).

### Case 3

A two-year six months-old male child underwent corrective surgery for developmental dysplasia of the hip. After the corrective surgery when the plastering of the limbs was being done in the operation theater he started having high spikes of temperature (106.6°F) followed by tachycardia, tachypnea, convulsions, and muscular rigidity. He was shifted to PICU, put on mechanical ventilation and emergency supportive management was started. Serum CPK came out to be 15200 U/L along with hyperkalemia and metabolic acidosis (pH 7.15, PCO_2_ 60 mm Hg, HCO_3_ 11 mEq/L). Hyperhydration with rapid correction of electrolyte and the acid–base balance was done. Whole body cooling was started with ice packs and cold saline infusion. The baby responded to treatment and was extubated after 48 hours of mechanical ventilation.

## DISCUSSION

Malignant hyperthermia (MH) is an uncommon but feared condition arising classically in genetically susceptible individuals after exposure to one or more of various triggering agents, most commonly a depolarizing muscle relaxant (succinylcholine) or an inhalational anesthetic agent (halothane, desflurane, enflurane, etc.) and rarely various stress factors like heat and exercise.^3^ In our case series, halothane was used in all the three patients without succinylcholine (Table 1).

**Table 1:** Demographic and clinical profile of patients

	*Case 1*	*Case 2*	*Case 3*
Age	10 months	1 year	2.5 years
Sex	male	male	male
Halothane used	yes	yes	yes
Succinylcholine used	no	no	no
Previous exposure to halothane	no	yes	no
Development of symptoms following surgery	40 min	25 min	30 min
Highest temperature	107°F	105.8°F	106.6°F
Convulsions	yes	yes	yes
Hematuria	yes	yes	yes
Serum creatine phosphokinase (CPK)	15970 U/L	16400 U/L	15200 U/L
Disseminated intravascular coagulation	yes	no	no
Acute kidney injury	yes	no	no
Ventilated	yes	yes	yes
Duration of ventilation	21 days	14 hours	48 hours
Outcome	survived, severe global developmental delay	died	survived, no neuromotor deficit

Though an MH crisis may develop at first exposure to triggering agents, on average, patients require three anesthesias before the reaction occurs. The crisis is more commonly reported in male patients (M: F 2:1).^4,5^ Highest incidence is in young people, one study reported that children under 15 years of age comprised 52.1% of reactions.^6,7^ All the three children we came across were male, under three years of age, and for one of them, it was the second operation under general anesthesia (the one operated for Hirschsprung's disease).

The incidence of MH reactions ranges from 1:10,000 to 1:2,50,000 anesthesias.^8,9^ The prevalence of the genetic abnormalities determining susceptibility may be as high as 1 in 3,000 individuals.^10^ Also, not one, but multiple genetic mutations have been identified to play roles in the causation.^11^ Lack of phenotypic expression without anesthesia makes it impossible to diagnose susceptibility without either the exposure to the “trigger” anesthetics or by specific diagnostic testing. However, due to unavailability genetic testing could not be performed in any of our patients.

In almost all cases of MH, defect lies in the ryanodine receptors in the sarcoplasmic reticulum of skeletal muscle, which acts as a cellular calcium channel.^12,13^ Abnormal receptor somehow barely maintains intracellular calcium homeostasis when not exposed to triggering agents. Once triggering agents stimulate calcium release, there is a vicious cycle resulting in a continuous increase in intracellular ionic calcium, which stimulates muscular contraction leading to a hypermetabolic state,

oxygen consumption, carbon dioxide production, ATP breakdown, and heat. Once the homeostatic mechanisms become exhausted, there is a decline in ATP level and failure to maintain membrane integrity. Loss of intracellular electrolytes (potassium, magnesium, phosphate) ensues followed by leaking out of myoglobin and creatine kinase leading to hyperkalemia and other dyselectrolytemia. There is a shutdown of the oxidative metabolism, formation of lactate, and ensuing acidosis stimulates sympathetic innervation, resulting in tachycardia, high blood pressure, and vasoconstriction.^14,15^ This dramatic rise in metabolic rate and oxygen consumption, if not treated promptly and effectively, results in the majority of cases in the patient's death.

An MH may occur during anesthesia or in the immediate postoperative period, but usually not after an hour of discontinuation of inhalational anesthetics.^16^ Two of our patients developed the crisis in the recovery room within 1 hour of surgery, and one in the operation theatre 25 minutes after surgery.

The clinical grading scale that has been used for diagnosis was developed by Larach et al. The criteria include components indicative of rigidity (general muscular rigidity, succinylcholine-induced masseter spasm), muscle breakdown (CPK > 20,000 after succinylcholine or >10,000 without, cola-colored urine, urine myoglobin >60 µg/L, serum myoglobin >170 µg/L, serum K^+^ >6 mEq/L in the absence of renal failure), respiratory acidosis (PET_CO_2__ >55 mm Hg/ arterial Pa_CO_2__ >60 mm Hg with appropriate controlled ventilation, PET_CO_2__ >60 mm Hg/ arterial Pa_CO_2__ >65 mm Hg with spontaneous ventilation, inappropriate hypercarbia or tachypnea), temperature increase (temperature >38.8° C or inappropriately increased temperature in anesthesiologist's judgment in the immediate postoperative period) and cardiac involvement (ventricular tachycardia, ventricular fibrillation or inappropriate sinus tachycardia).^17^ All of our patients have fulfilled these criteria to be labeled as MH.

Complications of malignant hyperthermia include cardiac dysfunction, pulmonary edema, renal failure, coma, disseminated intravascular coagulation (DIC), hepatic dysfunction, compartment syndrome, and prolonged intubation.^18^ One of our patients developed renal dysfunction, DIC and multiple infarcts in the brain.

Management of MH crisis consists of stopping all potent inhalational anesthetics and/or succinylcholine, increasing minute ventilation to lower ET_CO_2__, external cooling by ice packs, intravenous infusion of normal saline at 4°C, treatment of arrhythmias (amiodarone drug of choice; avoid calcium channel blockers), treatment of hyperkalemia and ensuring urine output of at least 2 mL/kg/hour with iv fluid, mannitol and furosemide.

The patient should be tended to in an intensive care unit. Dantrolene sodium is the only specific drug for the situation; the initial dose is 2.5 mg/kg iv, to be repeated every 10 to 15 minutes until acidosis, pyrexia, and muscle rigidity are resolving. Thereafter it has to be continued at a dose of 1 mg/kg every 4–8 hours for 24–48 hours.^19^ Sadly, Dantrolene was not available in our part of the country. Hence, we could only provide supportive treatment.

Mutational analysis or other diagnostic tests could not be performed in our patients. We believe the episodes were caused due to MH as suggested by the presence of classical clinical features and the rapidity with which they developed following surgical procedure under anesthesia with halothane.
